# Supplementary material for: Transcriptome analysis identified long non-coding RNAs involved in the adaption of yak to high-altitude environments
Source: R Soc Open Sci. 2020 Sep 23;7(9):200625. doi: 10.1098/rsos.200625 (PMC7540768; doi:10.1098/rsos.200625)
Supplement: supplementary files.docx [file rsos200625supp1.docx]

**Supplementary information:**

**Transcriptome analysis identified long non-coding RNAs involved in the adaption of yak to high-altitude environments**

Jin-Wei Xin^1,2,#^, Zhi-Xin Chai^3,#^, Cheng-Fu Zhang^1,2,#^, Yu-Mei Yang^3^, Qiang Zhang^1,2^, Yong Zhu^1,2^, Han-Wen Cao^1,2^, CidanYangJi^1,2^, Jin-Cheng Zhong^3^, Qiu-Mei Ji^1,2,*^

^1^. State Key Laboratory of Hulless Barley and Yak Germplasm Resources and Genetic Improvement, Lhasa, P. R. China

^2^. Institute of Animal Science and Veterinary, Tibet Academy of Agricultural and Animal Husbandry Sciences, Lhasa, P. R. China

^3^. Key Laboratory of Qinghai-Tibetan Plateau Animal Genetic Resource Reservation and Utilization, Sichuan Province and Ministry of Education, Southwest Minzu University, Chengdu, P. R. China

# J-W Xin, Z-X Chai and C-F Zhang contributed equally to this work.

* Corresponding author: Q-M Ji, E-mail: jiqiumei07@163.com, Tel: +86-13989908200

**Table S1. Primer sequences for real-time quantitative PCR used in the present study.**

| Gene ID | Forward (5’-3’) | Reverse (5’-3’) |
| --- | --- | --- |
| *β-actin* | GTGGCCGAGGACTTTGATTG | CCTGTAACAACGCATCTCATATT |
| MSTRG.119.1 | ACTTCAGGCAGTGGCTTCTT | GTGCACCCTGAACTCAAAGG |
| MSTRG.1490.1 | ACCTACCAAACCACAGCAGG | TGGAGGGAGGCCAACATACT |
| MSTRG.1880.1 | ACCTCACTGACCCCTGGATT | ATCTTTGCTGGGCAGCTAGT |
| MSTRG.3667.1 | GAACTGGGGAAGAGGGGAAC | CTTTGGGTCCAGTCAGTGGT |
| MSTRG.8120.1 | AACCCTGTCCAGAATGGACTC | CTGGTTTCCCTTACCGCACT |
| MSTRG.9611.1 | GTCCATCTCTGCTCCAACCG | CCTCTTCTCCACACAGACCAG |
| MSTRG.11072.2 | AGCTGGCCCTTCCTAGTGAT | GCACTCACGTCCTGTGACAT |
| MSTRG.11065.1 | CCCTAGCCTTCTCAGAATGACC | AGAAAGTGGGGCTACATTGAC |
| MSTRG.11390.3 | AGCATTCATCTGACCCAGCC | CCATCTCCGTGGGTTGGTTT |
| MSTRG.11413.24 | CTGATTCAACAGGCACACCAG | ATCCCATGTGGTTACTAGACTCATC |

**Table S2.** **Summary of clean data of transcriptome results.** SC: Sanjiang cattle; HC: Holstein cattle; TC: Tibetan cattle.

| Sample ID | Total reads | Total bases | GC content | Q20 | Q30 |
| --- | --- | --- | --- | --- | --- |
| SC-1 | 94,013,682 | 14,102,052,300 | 49.00% | 97.59% | 93.89% |
| SC-2 | 96,379,352 | 14,456,902,800 | 51.62% | 97.39% | 93.45% |
| SC-3 | 97,068,214 | 14,560,232,100 | 49.87% | 97.35% | 93.39% |
| HC-1 | 75,373,578 | 11,306,036,700 | 51.79% | 96.89% | 92.12% |
| HC-2 | 82,736,520 | 12,410,478,000 | 50.25% | 96.94% | 92.26% |
| HC-3 | 89,105,810 | 13,365,871,500 | 53.26% | 96.71% | 91.73% |
| TC-1 | 95,942,662 | 14,391,399,300 | 49.45% | 97.47% | 93.64% |
| TC-2 | 170,834,982 | 25,625,247,300 | 54.23% | 96.27% | 90.96% |
| TC-3 | 87,280,876 | 13,092,131,400 | 50.96% | 97.11% | 92.74% |
| Yak-1 | 88,494,718 | 13,274,207,700 | 50.56% | 97.10% | 92.92% |
| Yak-2 | 83,919,480 | 12,587,922,000 | 49.32% | 97.14% | 92.89% |
| Yak-3 | 81,092,188 | 12,163,828,200 | 52.53% | 96.73% | 91.98% |

**Table S3. Sample wise alignment percentage.**

| Sample ID | Paired reads | Unique-mapped | Multiple-mapped | Alignment |
| --- | --- | --- | --- | --- |
| SC-1 | 46589542 | 88.27% | 7.62% | 95.89% |
| SC-2 | 47640536 | 79.8% | 15.17% | 94.97% |
| SC-3 | 48274836 | 84.75% | 10.65% | 95.4% |
| HC-1 | 37480305 | 84.41% | 10.51% | 94.92% |
| HC-2 | 41162398 | 87.11% | 8.08% | 95.19% |
| HC-3 | 44286684 | 80.91% | 12.75% | 93.66% |
| TC-1 | 47532223 | 82.58% | 13.41% | 95.99% |
| TC-2 | 84605959 | 77.88% | 8.16% | 86.04% |
| TC-3 | 43149174 | 81.98% | 12.94% | 94.92% |
| Yak-1 | 43795172 | 86.49% | 9.07% | 95.56% |
| Yak-2 | 41355868 | 88.53% | 7.62% | 96.15% |
| Yak-3 | 40099831 | 81.23% | 12.98% | 94.21% |

**Table S4. Statistics of novel lncRNAs.**

| Index | Number or length |
| --- | --- |
| Total number | 1,364 |
| N10 (bp) | 7,523 |
| N20 (bp) | 5,514 |
| N30 (bp) | 4,374 |
| N40 (bp) | 3,064 |
| N50 (bp) | 2,342 |
| Median length (bp) | 883 |

**Table S5. Table 3. Selected genes targeted by DE lncRNAs in relation to energy metabolism and muscle contraction.** HC: Holstein cattle; SC: Sanjiang cattle; TC: Tibetan cattle. Cor: Pearson Correlation Coefficient. Data show mean ± standard error of FPKM.

| LncRNA | HC | SC | TC | Yak | Unigene ID | Description | HC | SC | TC | | Yak | Cor |
| --- | --- | --- | --- | --- | --- | --- | --- | --- | --- | --- | --- | --- |
| Energy metabolism | | | | | | | | | | | | |
| MSTRG.24686.4 | 92.6 ± 28.9 | 32.3 ± 15.3 | 23.8 ± 14.7 | 13.4 ± 5.3 | BmuPB000082 | Glyceraldehyde 3-phosphate dehydrogenase | 443.3 ± 122.7 | 200.0 ± 20.5 | 257.7 ± 126.8 | | 83.7 ± 2.6 | 0.926 |
| MSTRG.24686.4 | 92.6 ± 28.9 | 32.3 ± 15.3 | 23.8 ± 14.7 | 13.4 ± 5.3 | BmuPB012923 | Phosphoglycerate mutase 1 | 81.3 ± 23.9 | 24.0 ± 8.8 | 42.7 ± 6.6 | | 5.3 ± 4.2 | 0.908 |
| MSTRG.11882.2 | 43.9 ± 3.1 | 11.0 ± 8.8 | 4 ± 2.2 | 0.8 ± 0.4 | BmuPB021081 | Lactate/malate dehydrogenase | 8835.3 ± 2805.3 | 3281.3 ± 1106.2 | 4527.0 ± 1026.4 | | 1518.0 ± 239.0 | 0.907 |
| MSTRG.19579.8 | 55.8 ± 10.3 | 138.3 ± 32.6 | 60.9 ± 11.5 | 252.7 ± 42.4 | BmuPB015183 | Glyoxalase | 2255.7 ± 309.9 | 3599.3 ± 512.1 | 3349.7 ± 644.6 | | 3110.3 ± 639.5 | 0.959 |
| MSTRG.23564.4 | 5.5 ± 1.5 | 10.2 ± 1.2 | 5.5 ± 0.6 | 13.1 ± 1.2 | BmuPB001571 | Rieske iron-sulphur protein | 7452.0 ± 2959.8 | 11148.7 ± 2064.6 | 10021.3 ± 1997.9 | | 25730.3 ± 3414.1 | 0.952 |
| MSTRG.13013.1 | 4.3 ± 2.1 | 10.0 ± 0.9 | 5.1 ± 1.3 | 10.8 ± 1.4 | BmuPB001888 | Glycoside hydrolase | 1704.0 ± 618.3 | 3486.3 ± 801.7 | 3622.7 ± 581.9 | | 5216.7 ± 223.6 | 0.946 |
| MSTRG.13013.1 | 4.3 ± 2.1 | 10.0 ± 0.9 | 5.1 ± 1.3 | 10.8 ± 1.4 | BmuPB003333 | Sodium: alanine symporter | 6508.7 ± 3359.2 | 12698.7 ± 5405.7 | 13266.0 ± 2155.7 | | 22812.3 ± 1443.1 | 0.911 |
| MSTRG.21768.2 | 2.5 ± 1.5 | 2.7 ± 0.9 | 4.5 ± 1.9 | 5.0 ± 1.5 | BmuPB004383 | Acyl carrier protein | 2114.0 ± 753.4 | 2546.3 ± 516.6 | 2743.3 ± 686.7 | | 6336.3 ± 971.6 | 0.916 |
| MSTRG.26388.4 | 73.4 ± 26.4 | 126.2 ± 22.7 | 81.4 ± 20.0 | 144.2 ± 18.6 | BmuPB010683 | Orphan nuclear receptor | 989.7 ± 423.2 | 2044.0 ± 593.5 | 1812.0 ± 453.1 | | 2979.3 ± 676.4 | 0.960 |
| MSTRG.12635.1 | 3.7 ± 1.3 | 6.0 ± 0.9 | 4.6 ± 2.6 | 0.1 ± 0.1 | BmuPB004072 | Peptidase M16 | 532.3 ± 224.3 | 944.3 ± 304.8 | 966.0 ± 394.1 | | 233.3 ± 158.9 | 0.917 |
| MSTRG.5970.1 | 5.4 ± 4.8 | 0.6 ± 0.4 | 0.8 ± 1.0 | 0.3 ± 0.4 | BmuPB005211 | ATP synthase | 3439.0 ± 2431.7 | 1528.0 ± 245.8 | 1953.7 ± 414.1 | | 1489.7 ± 78.3 | 0.916 |
| MSTRG.23993.3 | 19.0 ± 0.9 | 14.4 ± 2.7 | 9.2 ± 1.5 | 1.4 ± 0.1 | BmuPB009505 | NADH dehydrogenase | 35.7 ± 14.1 | 34.7 ± 6.8 | 26.7 ± 4.2 | | 3.3 ± 2.1 | 0.926 |
| MSTRG.18165.1 | 1.0 ± 0.8 | 1.2 ± 0.2 | 2.4 ± 0.4 | 2.8 ± 0.6 | BmuPB006563 | Glycoside hydrolase | 6.7 ± 3.7 | 11.7 ± 9.7 | 57.3 ± 8.0 | | 29.0 ± 6.4 | 0.915 |
| MSTRG.21768.2 | 2.5 ± 1.5 | 2.7 ± 0.9 | 4.5 ± 1.9 | 5.0 ± 1.5 | BmuPB019813 | Aminotransferase | 566.7 ± 188.7 | 651.3 ± 311.9 | 1141.3 ± 457.8 | | 1804.7 ± 500.3 | 0.922 |
| MSTRG.16170.2 | 9.4 ± 4.8 | 20.9 ± 7.2 | 10.9 ± 1.7 | 20.0 ± 3.2 | BmuPB002483 | Eukaryotic molybdopterin oxidoreductase | 3250.7 ± 569.4 | 6165.7 ± 1968.7 | 6009.7 ± 1143.9 | | 9205.7 ± 818.2 | 0.905 |
| MSTRG.16170.2 | 9.4 ± 4.8 | 20.9 ± 7.2 | 10.9 ± 1.7 | 20.0 ± 3.2 | BmuPB003110 | Acyl-CoA oxidase/dehydrogenase | 8310.3 ± 2564.2 | 14716.3 ± 2997.6 | 18485 ± 3807.3 | | 24381.7 ± 4024.0 | 0.913 |
| MSTRG.23564.4 | 5.5 ± 1.5 | 10.2 ± 1.2 | 5.5 ± 0.6 | 13.1 ± 1.2 | BmuPB003734 | Porin, Oms28 type | 4545.7 ± 1278.7 | 5133.7 ± 1233.6 | 8611.3 ± 878.2 | | 15076.0 ± 3536.0 | 0.905 |
| MSTRG.13013.1 | 4.3 ± 2.1 | 10 ± 0.9 | 5.1 ± 1.3 | 10.8 ± 1.4 | BmuPB008201 | Acyl-CoA-binding protein | 2369.0 ± 550.4 | 4779.0 ± 2014.1 | 3676.0 ± 1140.2 | | 7804.0 ± 1801.6 | 0.915 |
| MSTRG.23394.1 | 1.1 ± 0.1 | 2.2 ± 1.0 | 3.7 ± 1.3 | 3.8 ± 1.3 | BmuPB012824 | Acyl-CoA oxidase/dehydrogenase | 7738.3 ± 1863.3 | 18113.7 ± 6793.8 | 16825.0 ± 3938.5 | | 26469.0 ± 3783.5 | 0.906 |
| MSTRG.26388.4 | 73.4 ± 26.4 | 126.2 ± 22.7 | 81.4 ± 20.0 | 144.2 ± 18.6 | BmuPB017956 | Acyl-CoA oxidase/dehydrogenase | 881.3 ± 233.9 | 1475.7 ± 580.3 | 1762.0 ± 425.1 | | 2895.3 ± 876.2 | 0.914 |
| MSTRG.26388.4 | 73.4 ± 26.4 | 126.2 ± 22.7 | 81.4 ± 20.0 | 144.2 ± 18.6 | BmuPB019952 | Crotonase superfamily | 2368.3 ± 860.7 | 5532.0 ± 1691.9 | 3901.3 ± 817.2 | | 7200.7 ± 1319.4 | 0.928 |
| MSTRG.26388.4 | 73.4 ± 26.4 | 126.2 ± 22.7 | 81.4 ± 20.0 | 144.2 ± 18.6 | BmuPB021264 | 3-hydroxyacyl-CoA dehydrogenase | 1562.7 ± 216.2 | 2949.7 ± 744.4 | 2624.7 ± 1150.2 | | 4474.7 ± 807.9 | 0.913 |
| MSTRG.24686.4 | 92.6 ± 28.9 | 32.3 ± 15.3 | 23.8 ± 14.7 | 13.4 ± 5.3 | BmuPB009427 | Pyruvate kinase | 87848.3 ± 25949.6 | 34413.7 ± 13983.9 | 40381.3 ± 6894.2 | | 40991.3 ± 9959.0 | 0.920 |
| MSTRG.24686.4 | 92.6 ± 28.9 | 32.3 ± 15.3 | 23.8 ± 14.7 | 13.4 ± 5.3 | BmuPB017171 | Lactate/malate dehydrogenase | 88118.3 ± 26408.2 | 36308.0 ± 18699.9 | 40240.0 ± 9328.7 | | 36478.3 ± 4908.4 | 0.924 |
| MSTRG.21891.1 | 3.4 ± 0.3 | 18.0 ± 17.6 | 12.4 ± 5.5 | 20.3 ± 16.0 | BmuPB008988 | GNS1/SUR4 membrane protein | 55.3 ± 17.5 | 335.0 ± 382.7 | 348.7 ± 131.2 | | 384.7 ± 319.2 | 0.913 |
| Muscle contraction | | | | | | | | | | | | |
| MSTRG.2086.1 | 1.1 ± 1.5 | 0.0 ± 0.0 | 1.2 ± 0.7 | 5.5 ± 1.0 | BmuPB011617 | EF-hand domain | 12440.7 ± 12892.0 | 8904.3 ± 5938.0 | 30180.7 ± 3115.9 | | 61758.7 ± 10735.2 | 0.931 |
| MSTRG.11372.1 | 32.4 ± 6.9 | 33.3 ± 20.0 | 7.2 ± 2.3 | 4.8 ± 1.7 | BmuPB001050 | Calcium-release channel | 148606.3 ± 22423.4 | 163420.0 ± 81301.1 | 105313.3 ± 18278.5 | | 81564.0 ± 21368.5 | 0.909 |
| MSTRG.1770.1 | 1.3 ± 0.9 | 8.5 ± 6.1 | 3.8 ± 1.5 | 1.0 ± 0.4 | BmuPB019192 | Tropomyosin | 1029.7 ± 397.7 | 3958.0 ± 2623.3 | 2789.3 ± 309.7 | 21.3 ± 4.8 | | 0.946 |
| MSTRG.11409.1 | 10.8 ± 3.9 | 14.0 ± 11.2 | 6.8 ± 4.5 | 2.2 ± 1.2 | BmuPB017752 | Titin | 211263.7 ± 71209.6 | 273364.3 ± 151374 | 158144.7 ± 11520.6 | | 115022.3 ± 24256.2 | 0.913 |
| MSTRG.23564.4 | 5.5 ± 1.5 | 10.2 ± 1.2 | 5.5 ± 0.6 | 13.1 ± 1.2 | BmuPB001571 | Rieske iron-sulphur protein | 7452.0 ± 2959.8 | 11148.7 ± 2064.6 | 10021.3 ± 1997.9 | | 25730.3 ± 3414.1 | 0.952 |
| MSTRG.25261.1 | 2.9 ± 0.6 | 16.5 ± 10.5 | 3.1 ± 1.2 | 18.5 ± 6.3 | BmuPB009477 | Myosin | 245493.7 ± 103617.0 | 743940.3 ± 434993.7 | 583485.7 ± 116049.2 | | 757539.3 ± 34933.3 | 0.916 |
| MSTRG.16892.1 | 7.1 ± 0.8 | 5.8 ± 1.4 | 1.7 ± 0.8 | 1.5 ± 0.3 | BmuPB003310 | Sarcoglycan | 2929.0 ± 279.4 | 2364.0 ± 528.5 | 1921.3 ± 256.8 | | 1843.3 ± 172.8 | 0.911 |
| MSTRG.24686.4 | 92.6 ± 28.9 | 32.3 ± 15.3 | 23.8 ± 14.7 | 13.4 ± 5.3 | BmuPB010503 | Tropomyosin | 447035.0 ± 151025.2 | 219115.3 ± 110770.5 | 182749.3 ± 14838.7 | | 162203.3 ± 53871.9 | 0.929 |
| MSTRG.16892.1 | 7.1 ± 0.8 | 5.8 ± 1.4 | 1.7 ± 0.8 | 1.5 ± 0.3 | BmuPB010641 | Voltage-dependent calcium channel | 31676.7 ± 3781.2 | 23626.7 ± 2906.0 | 20961.7 ± 2019.8 | | 18534.3 ± 1590.5 | 0.904 |
| MSTRG.21048.1 | 15.9 ± 8.5 | 0.2 ± 0.2 | 1.3 ± 0.8 | 0.1 ± 0.1 | BmuPB011717 | Voltage-dependent calcium channel | 349.7 ± 193.4 | 35.3 ± 40.2 | 39.0 ± 24.9 | | 68.7 ± 22.4 | 0.958 |
| MSTRG.12635.1 | 3.7 ± 1.3 | 6.0 ± 0.9 | 4.6 ± 2.6 | 0.1 ± 0.1 | BmuPB004072 | Peptidase M16 | 532.3 ± 224.3 | 944.3 ± 304.8 | 966.0 ± 394.1 | | 233.3 ± 158.9 | 0.917 |
